# Short-term outcomes in patients with systemic juvenile idiopathic arthritis treated with either tocilizumab or anakinra

**DOI:** 10.1093/rheumatology/key262

**Published:** 2018-08-21

**Authors:** Lianne Kearsley-Fleet, Michael W Beresford, Rebecca Davies, Diederik De Cock, Eileen Baildam, Helen E Foster, Taunton R Southwood, Wendy Thomson, Kimme L Hyrich

**Affiliations:** 1 Arthritis Research UK Centre for Epidemiology, Manchester Academic Health Science Centre, The University of Manchester, Manchester, UK; 2 Institute of Translational Medicine (Child Health), University of Liverpool, UK; 3 Clinical Academic Department of Paediatric Rheumatology, Alder Hey Children’s NHS Foundation Trust, Liverpool, UK; 4 Institute of Cellular Medicine, Newcastle University, Newcastle upon Tyne, UK; 5 Paediatric Rheumatology, Great North Children’s Hospital, Newcastle upon Tyne, UK; 6 Institute of Child Health, University of Birmingham and Birmingham Children’s Hospital, Birmingham, UK; 7 Arthritis Research UK Centre for Genetics and Genomics, Manchester Academic Health Science Centre, The University of Manchester, Manchester, UK; 8 National Institute of Health Research Manchester Biomedical Research Centre, Manchester Academic Health Science Centre, Manchester University NHS Foundation Trust, Manchester, UK

**Keywords:** JIA, biologic therapies, epidemiology, outcome measures, statistics

## Abstract

**Objectives:**

To investigate real-world short-term outcomes among patients with systemic JIA starting tocilizumab or anakinra.

**Methods:**

This analysis included all systemic JIA patients within the UK Biologics for Children with Rheumatic Diseases study starting tocilizumab or anakinra between 2010 and 2016. Disease activity was assessed at baseline and one year. At one year the following outcomes were assessed: minimal disease activity, clinically inactive disease, 90% ACR Paediatric response (ACRPedi90). Univariable logistic regression was used to identify baseline characteristics associated with these outcomes. Multiple imputation was used to account for missing data.

**Results:**

Seventy-six systemic JIA patients were included (54 tocilizumab; 22 anakinra). More patients starting anakinra as their first biologic compared with tocilizumab (86% *vs* 63%; *P* = 0.04), with shorter disease duration (1 *vs* 2 years; *P* = 0.003) and higher frequency of prior macrophage activation syndrome (37% *vs* 8%; *P* = 0.004). Overall, at one year, 42% achieved ACRPedi90, 51% minimal disease activity, and 39% clinically inactive disease, with similar responses seen between the two drugs. Response was not associated with baseline disease characteristics. Fifteen (20%) patients stopped biologic treatment by one year. Treatment survival was better with tocilizumab (89% at one year *vs* 59% anakinra; *P* = 0.002), with three stopping for anakinra injection-related problems.

**Conclusion:**

In this real-world cohort of patients with systemic JIA receiving tocilizumab or anakinra, approximately half achieved a minimal disease state by one year. Treatment responses appeared similar between the two therapies albeit with better persistence observed with tocilizumab.

**Rheumatology key messages**

- Tocilizumab and anakinra were effective treatments for systemic JIA; half achieved minimal disease activity.
- Treatment response appeared to be similar between systemic JIA patients treated with tocilizumab and anakinra.
- More systemic JIA patients remained on tocilizumab at one year; anakinra patients reported more injection-related problems.

## Introduction

JIA is a diagnosis of exclusion and represents arthritis that begins before a child turns sixteen years of age and persists for at least six weeks in which no other cause has been identified. It affects ∼3 in 10 000 children and young people [1]. The current international categorisation of the condition is the ILAR classification, which includes seven different categories [2]. While this classification was based predominantly on clinical characteristics, systemic JIA remains clinically distinct from the other ILAR categories of JIA, with systemic involvement including fever, rash and enlarged lymph nodes [3]. Recent genetic analysis has shown marked variation in the loci associated with systemic JIA compared with other JIA ILAR categories [4]. In addition, it has a markedly distinct underlying mechanism of disease, including an important role of the innate immune system. It is driven by specific pro-inflammatory cytokines (e.g. IL-1, IL-6) contributing to multisystem inflammation [5]. This knowledge has led to different treatment strategies for systemic JIA compared with other JIA ILAR categories, with a shift away from TNF inhibitors (TNFi) in favour of IL-6 pathway inhibitors (such as tocilizumab) and therapies that block IL-1 (such as anakinra or canakinumab) [6–8].

Patients with systemic JIA in the United Kingdom (UK) may be prescribed a biologic DMARD after failing or being intolerant to the conventional synthetic DMARD methotrexate. TNFi were previously prescribed as a first-line biologic therapy in all patients with JIA [9]. The 2015 National Health Service England treatment pathway [10] now recommends that patients with systemic JIA be prescribed tocilizumab (an IL-6 pathway inhibitor) or anakinra (IL-1 receptor antagonist) as a first biologic therapy following failure of methotrexate. The exception to this is children who present with macrophage activation syndrome (MAS) unresponsive to intravenous steroids, who should be treated with anakinra first-line.

Since 2010, there has been a shift in the UK towards the use of tocilizumab or anakinra as a first-line biologic following methotrexate in children with systemic JIA [11]. Tocilizumab is licenced for use in patients with systemic JIA following evidence of efficacy from clinical trials [7, 12–16]. Published evidence on the use of anakinra for JIA is limited. One small randomised controlled trial of only one month duration found evidence of benefit compared with placebo in patients with systemic JIA [6]. In a French retrospective study of 77 systemic JIA patients starting a first biologic (predominantly anakinra), approximately half had achieved and maintained inactive disease after a median of over two years of follow-up [17]. The majority of observational studies on anakinra are low in patient number [18–21]. A recent study from the German paediatric biologics register Biologika in der Kinderrheumatologie has investigated outcomes in patients with systemic JIA treated with either tocilizumab or an IL-1 receptor antagonist (anakinra or canakinumab). At one year, 27% and 35% of patients achieved an ACR paediatric 90% response (ACR Pedi 90) on tocilizumab and an IL-1 inhibitor respectively. There was no difference between the two drug cohorts with respect to remission or minimal disease activity using disease activity scores [22].

This analysis aimed to describe and compare the real-world therapeutic short-term outcomes among children and young people with systemic JIA starting either tocilizumab or anakinra in order to create an evidence base to inform clinicians about the use of these agents in clinical practice. The objectives of this analysis were to (1) investigate and compare baseline characteristics in all children and young people in the UK between 2010 and 2016 starting either tocilizumab or anakinra for systemic JIA, (2) measure and compare short-term outcomes, including treatment response, treatment survival and stop reasons by one year of treatment between children starting (a) tocilizumab *vs* anakinra, and (b) either tocilizumab or anakinra as a first-line *vs* subsequent-line biologic therapy, and (3) investigate associations between baseline characteristics and outcomes at one year.

## Methods

### Study setting, data capture and study population

This analysis used data collected from the UK’s Biologics for Children with Rheumatic Diseases (BCRD) study [11]. This register, established in 2010, captures data about children and young people with JIA starting a biologic therapy other than Enbrel (etanercept); patients starting Enbrel are recruited to an alternative study in the UK [23]. Patients are recruited to the study at the point of starting a new biologic therapy but do not have to be biologic naïve. Nationally, recruitment is recommended [10] but not mandatory. The study was approved by the North West 7 REC Greater Manchester Central Ethics Committee. Written informed consent was obtained from all parents (or patients where appropriate) in accordance with the Declaration of Helsinki. Additional ethical approval to analyse these data was not required.

At registration, the start of biologic therapy, the treating physician or affiliated clinical research nurse completed a detailed questionnaire on patient demographics, disease characteristics, ILAR classification and disease activity, and all current and past anti-rheumatic therapies, including prior biologics, and other medications. Follow-up questionnaires were completed at six months, one year and then annually thereafter. Details of changes to drug therapy, as well as current disease activity measures, were documented. The occurrence of any adverse events or new health diagnoses were recorded.

Patients with systemic JIA registered starting either tocilizumab or anakinra from 1 January 2010 with baseline and one year data returned before 31 December 2016 were included in this study. Baseline disease characteristics were assessed; including the 71-joint juvenile arthritis disease activity score (JADAS-71) [24]. Patients were excluded if they were in minimal disease activity (MDA) [25] at the start of biologic therapy with no systemic features present (*n* = 2). For logistical reasons, patients could be registered into the study within six months of starting biologic therapy, although it was requested in all cases that data entered into the study database was that reflecting the start of therapy and not current measures at the point of registration. As it was felt that these cases with very low disease activity at the start of therapy were highly unlikely to be correct, it was assumed their data were recorded after the drug had been started and therefore these cases were excluded.

### Analyses

#### Baseline characteristics

The baseline characteristics were compared between patients starting anakinra *vs* tocilizumab. Categorical baseline characteristics were compared used Pearson’s chi-squared test, and continuous variables were compared between groups using nonparametric K-sample test on the equality of medians. Baseline characteristics were also compared between patients starting either drug as a first-line biologic *vs* patients who had prior biologic exposure.

#### Primary outcomes

Three primary outcome measures were investigated at one year after start of biologic; proportion achieving MDA [25], proportion achieving clinically inactive disease (CID) [26], and proportion achieving ACR Pedi 90 response [27]. Both the MDA and CID criteria assess disease activity at a single time point. Patients with systemic JIA were defined as achieving MDA if the physician global assessment of disease activity (PGA) was no >3.4 cm, the patient (or parent) global evaluation of well-being (PGE) was no >2.1 cm, with a maximum of one active joint [25]. Patients were defined as achieving CID if they had no active joints, no systemic features, no active uveitis, PGA of zero, and a normal ESR defined in this study as 20 mg/mm or less [26]. The ACR paediatric response criterion assesses change in disease activity over time and can be assessed with differing percentages of achievement. A patient was defined as achieving an ACR Pedi 90 if three of the six JIA core outcome variables (active joint count, limited joint count, PGA, PGE, childhood HAQ (CHAQ) for functional ability, and ESR) improved by at least 90%, with a maximum of one variable worsening by >30% [27]. Patients with a baseline core outcome variable of zero who worsen over time were classified to worsen that variable by >30%. Patients who improved core outcome variable down to zero over time were classified to improve that variable by 100%. Patients with a baseline core outcome variable of zero and remained at zero over time improved by 0% (neither improved nor worsened). Patients who stopped biologic therapy before one year were classified as failing to achieve these outcomes, unless the stop reason was remission, in which case they were classified as achieving all outcomes.

Primary outcomes were compared between patients starting tocilizumab *vs* anakinra, and also between patients starting anakinra or tocilizumab as a first-line biologic *vs* patients starting as a subsequent biologic therapy. Statistical significance between cohorts was assessed using logistic regression. In addition, the logistic regression was adjusted using a propensity score to compare outcomes in patients treated with tocilizumab *vs* anakinra. The propensity score included: whether the patient was starting it as a first-biologic, gender, age, disease duration, concomitant methotrexate use, concomitant steroid use, active joint count, limited joint count, PGA, PGE, CHAQ, ESR and JADAS-71.

#### Secondary outcomes

Secondary effectiveness outcomes studied included the change in active joint count, limited joint count, PGA, PGE, CHAQ, ESR and JADAS-71, using regression models adjusted for baseline values. A drug survival analysis was performed using a Kaplan-Meier curve to present the proportion of patients who stopped biologic therapy by one year. The stop reasons of therapy given by the treating physician were categorised and described for each drug cohort: inefficacy, remission, adverse event.

Secondary effectiveness outcomes and drug survival were compared between patients starting tocilizumab *vs* anakinra, and between patients starting either drug as first-line biologic *vs* subsequent biologic. Statistical significance between cohorts was assessed using logistic regression for secondary effectiveness outcomes and a log-rank test for equality of survivor functions for the drug survival. Univariable logistic regression was used to assess the associations of baseline characteristics with the primary outcomes at one year, including patient characteristics (age, gender), disease features (disease duration, methotrexate use, steroid use), disease activity (including core outcome variables), and choice of treatment (tocilizumab *vs* anakinra).

Multiple imputation (with 80 iterations based on proportion of incomplete cases [28]) was used to account for missing data. Complete variables included biologic therapy (anakinra or tocilizumab), whether the patient was starting it as a first-biologic, age at biologic start, gender, concomitant methotrexate use, concomitant steroid use, discontinuation of biologic in the first year (not for remission). Imputed values included disease duration at start of biologic, disease activity measures at the start of therapy and at one year (active joint count, limited joint count, PGA, PGE, CHAQ, ESR) and whether patient had systemic features at one year. From the imputed values, the outcome variables could be calculated: JADAS-71 (at baseline and one year), change in JADAS-71 from baseline, change in CHAQ from baseline, MDA at one year, CID at one year, and ACR Pedi 90 response at one year. Stata version 13 was used to perform all analyses [29].

## Results

A total of 76 patients had registered fulfilling the ILAR criteria for systemic JIA: 54 starting tocilizumab, 22 starting anakinra. Table 1 shows the baseline characteristics. In total, 57% were female, and 70% were starting a biologic for the first time. The majority of patients had prior exposure to methotrexate: 98% of tocilizumab and 86% of anakinra (*P* = 0.04). Of the patients who had previously used a biologic therapy, 39% had used two or more. The majority of patients with prior biologic exposure had used a TNFi (78%), with 11 of the 20 tocilizumab patients (55%) previously exposed to an IL-1 inhibitor, and two of the three anakinra patients (67%) previously exposed to tocilizumab. Median age at start of registered biologic was seven years old (six years for first-line biologic patients, nine years old otherwise; *P* = 0.07), and median disease duration from diagnosis to biologic treatment was one year (one year for first-line biologic users, three years otherwise; *P* < 0.001). Approximately 59% of patients had systemic features present when starting either tocilizumab or anakinra and 16% had a history of MAS.

**Table 1:** Baseline characteristics of all patients with systemic JIA starting either tocilizumab or anakinra at registration

	All patients *n* = 76	Tocilizumab *n* = 54	Anakinra *n* = 22	*P*-value (tocilizumab *vs* anakinra)	First biologic *n* = 53	Subsequent biologic *n* = 23	*P*-value (first biologic *vs* subsequent)
Female	43 (57)	28 (52)	15 (68)	*P* = 0.2	27 (51)	16 (70)	*P* = 0.1
Tocilizumab	54 (71)	—	—	—	34 (64)	20 (87)	*P* = 0.04
First biologic	53 (70)	34 (63)	19 (86)	*P* = 0.04	—	—	—
Previous biologics	*n* = 23	*n* = 20	*n* = 3	*P* = 0.2	—	—	—
One previous	14 (61)	12 (60)	2 (67)	—	—	—	—
Two previous	7 (30)	6 (30)	1 (33)	—	—	—	—
Three previous	2 (9)	2 (10)	—	—	—	—	—
Biologic history	*n* = 23	*n* = 20	*n* = 3	—	—	—	—
TNF inhibitors (etanercept, infliximab, and adalimumab)	18 (78)	16 (80)	2 (67)	—	—	—	—
IL-1 inhibitors (anakinra and canakinumab)	11 (48)	11 (55)	0	—	—	—	—
IL-6 inhibitors (tocilizumab)	2 (9)	0	2 (67)	—	—	—	—
Age, median (IQR), years	7 (3–12)	7 (4–11)	6 (2–13)	*P* = 1.0	6 (3–11)	9 (5–14)	*P* = 0.07
Disease duration, median (IQR), years	1 (0–3) *n* = 75	2 (1–3)	1 (0–1) *n* = 21	*P* = 0.003	1 (0–1) *n* = 52	3 (2–5)	*P* < 0.001
Systemic features present	35 (59) *n* = 59	24 (53) *n* = 45	11 (79) *n* = 14	*P* = 0.09	24 (60) *n* = 40	11 (58) *n* = 19	*P* = 0.9
MAS history	11 (16) *n* = 68	4 (8) *n* = 49	7 (37) *n* = 19	*P* = 0.004	8 (17) *n* = 46	3 (14) *n* = 22	*P* = 0.7
Prior methotrexate exposure	72 (95)	53 (98)	19 (86)	*P* = 0.04	49 (92)	23 (100)	*P* = 0.2
Concomitant methotrexate	63 (83)	44 (81)	19 (86)	*P* = 0.5	47 (89)	16 (70)	*P* = 0.04
Prior steroid exposure	75 (99)	53 (98)	22 (100)	*P* = 0.5	53 (100)	22 (96)	*P* = 0.1
Concomitant steroids	49 (64)	36 (67)	13 (59)	*P* = 0.5	37 (70)	12 (52)	*P* = 0.1
Disease activity, median (IQR)							
Active joint count, 71 joints	4 (1–9) *n* = 65	4 (1–8) *n* = 48	5 (1–11) *n* = 17	*P* = 0.8	3 (0–6) *n* = 45	7 (5–11) *n* = 20	*P* = 0.01
Limited joint count, 71 joints	3 (0–7) *n* = 66	3 (1–7) *n* = 48	3 (0–11) *n* = 18	*P* = 0.9	1 (0–5) *n* = 45	6 (3–10) *n* = 21	*P* = 0.001
PGA, 0–10 cm VAS	3 (2–6) *n* = 49	4 (2–6) *n* = 34	2 (2–6) *n* = 15	*P* = 0.6	3 (1–5) *n* = 33	5 (3–7) *n* = 16	*P* = 0.03
PGE, 0–10 cm VAS	4 (2–7) *n* = 50	4 (2–7) *n* = 34	4 (1–6) *n* = 16	*P* = 0.9	4 (1–6) *n* = 33	5 (3–7) *n* = 17	*P* = 0.8
CHAQ, range 0–3	0.9 (0.4–2.0) *n* = 47	0.9 (0.4–1.8) *n* = 34	1.1 (0.5–2.0) *n* = 13	*P* = 0.5	0.9 (0.4–2.0) *n* = 30	1.3 (0.5–1.6) *n* = 17	*P* = 0.5
Pain VAS, 0–10 cm VAS	4 (1–6) *n* = 46	4 (1–6) *n* = 32	4 (1–6) *n* = 14	*P* = 0.9	4 (1–6) *n* = 30	4 (1–6) *n* = 16	*P* = 0.6
ESR, mm/h	33 (10–63) *n* = 66	26 (10–58) *n* = 49	55 (27–86) *n* = 17	*P* = 0.3	26 (10–63) *n* = 45	40 (21–58) *n* = 21	*P* = 0.3
CRP, mm/h	37 (4–82) *n* = 71	18 (4–63) *n* = 53	64 (19–95) *n* = 18	*P* = 0.2	38 (4–82) *n* = 50	33 (5–70) *n* = 21	*P* = 0.9
JADAS-71	19 (7–27) *n* = 33	19 (6–30) *n* = 11	20 (11–26) *n* = 22	*P* = 0.9	11 (7–22) *n* = 21	25 (20–31) *n* = 12	*P* = 0.05

Unimputed data. Results presented as *n* (%) unless otherwise stated.

MAS: macrophage activation syndrome; PGA: physician global assessment of disease; PGE: patient (or parent) global evaluation of well-being; CHAQ: Childhood HAQ; VAS: visual analogue scale; JADAS-71: 71-joint Juvenile Arthritis Disease Activity Score; IQR: interquartile range.

At one year, 42% of patients had achieved an ACR Pedi 90, 51% had achieved MDA, and 39% CID (Table 2). Mean change in JADAS-71 from baseline to one year was –14 units (*P* < 0.001), and mean change in CHAQ was –0.5 units (*P* < 0.001). Twenty percent of the patients reported systemic features at one year. In the univariable logistic regression models no baseline clinical characteristics were associated with achieving any of the three primary outcomes (Supplementary Table S1, available at *Rheumatology* online), including no difference between the two drug cohorts (tocilizumab *vs* anakinra), nor between patients starting biologic as first-line therapy *vs* patients with reporting prior biologic use. In addition, there was no difference between tocilizumab and anakinra with regard to achieving any of the three primary outcomes when adjusted by the propensity score (Table 2).

**Table 2:** Outcomes in all patients with systemic JIA starting either tocilizumab or anakinra

		All patients *n* = 76	Tocilizumab *n* = 54	Anakinra *n* = 22	*P*-value (tocilizumab *vs* anakinra)	First biologic *n* = 53	Subsequent biologic *n* = 23	*P*-value (first biologic *vs* subsequent)
Systemic features, %	One year	20%	17%	27%	*P* = 0.3	21%	20%	*P* = 0.9
Active joint count	Baseline	6.8 (1.1)	6.7 (1.3)	7.1 (2.1)	*P* = 0.9	5.3 (1.1)	10.4 (2.3)	*P* = 0.03
	One year	0.6 (0.2)	0.5 (0.2)	0.8 (0.4)	*P* = 0.6	0.5 (0.2)	0.8 (0.4)	*P* = 0.4
	Change^a^	−6.2 (1.0) *P* < 0.001	−6.2 (1.2) *P* < 0.001	−6.4 (2.0) *P* < 0.001	*P* = 0.6	−4.8 (1.1) *P* < 0.001	−9.6 (2.2) *P* < 0.001	*P* = 0.8
Limited joint count	Baseline	5.5 (1.0)	5.1 (1.1)	6.3 (2.1)	*P* = 0.6	3.9 (1.0)	9.1 (2.2)	*P* = 0.02
	One year	1.0 (0.3)	1.0 (0.3)	1.1 (0.6)	*P* = 0.8	0.7 (0.3)	1.9 (0.7)	*P* = 0.06
	Change^a^	−4.4 (1.0) *P* < 0.001	−4.1 (1.1) *P* < 0.001	−5.2 (2.1) *P* < 0.001	*P* = 0.9	−3.2 (1.0) *P* < 0.001	−7.2 (2.2) *P* < 0.001	*P* = 0.2
PGA	Baseline	3.7 (0.4)	3.9 (0.4)	3.2 (0.7)	*P* = 0.4	3.3 (0.5)	4.6 (0.6)	*P* = 0.1
	One year	1.0 (0.3)	1.0 (0.3)	1.1 (0.5)	*P* = 0.9	1.1 (0.4)	1.0 (0.5)	*P* = 0.9
	Change^a^	−2.7 (0.5) *P* < 0.001	−2.9 (0.6) *P* < 0.001	−2.1 (0.9) *P* = 0.002	*P* = 0.9	−2.3 (0.6) *P* < 0.001	−3.6 (0.8) *P* = 0.002	*P* = 0.9
PGE	Baseline	4.3 (0.4)	4.3 (0.5)	4.1 (0.8)	*P* = 0.8	4.1 (0.5)	4.6 (0.7)	*P* = 0.6
	One year	1.9 (0.4)	1.9 (0.4)	2.0 (0.7)	*P* = 0.8	2.1 (0.4)	1.5 (0.5)	*P* = 0.5
	Change^a^	−2.4 (0.5) *P* < 0.001	−2.5 (0.6) *P* < 0.001	−2.1 (1.0) *P* = 0.005	*P* = 0.8	−2.1 (0.6) *P* < 0.001	−3.1 (0.9) *P* < 0.001	*P* = 0.4
CHAQ	Baseline	1.1 (0.1)	1.1 (0.2)	1.1 (0.3)	*P* = 1.0	1.1 (0.2)	1.1 (0.2)	*P* = 1.0
	One year	0.6 (0.1)	0.6 (0.1)	0.7 (0.2)	*P* = 0.7	0.7 (0.1)	0.5 (0.2)	*P* = 0.4
	Change^a^	−0.5 (0.1) (*P* < 0.001)	−0.5 (0.2) (*P* < 0.001)	−0.4 (0.2) (*P* = 0.005)	*P* = 0.6	−0.4 (0.2) (*P* < 0.001)	−0.6 (0.2) (*P* = 0.005)	*P* = 0.4
ESR	Baseline	42 (5)	37 (5)	54 (10)	*P* = 0.1	39 (6)	48 (8)	*P* = 0.4
	One year	7 (1)	5 (1)	11 (2)	*P* = 0.02	7 (1)	7 (2)	*P* = 0.9
	Change^a^	−35 (5) *P* < 0.001	−32 (5) *P* < 0.001	−43 (11) *P* < 0.001	*P* = 0.02	−33 (6) *P* < 0.001	−41 (8) *P* < 0.001	*P* = 0.9
JADAS-71	Baseline	17 (1.5)	17 (1.8)	18 (2.9)	*P* = 0.8	15 (1.6)	23 (3.1)	*P* = 0.03
	One year	4 (0.6)	4 (0.7)	4 (1.3)	*P* = 0.7	4 (0.7)	4 (1.0)	*P* = 0.09
	Change^a^	−14 (1.6) (*P* < 0.001)	−14 (1.8) (*P* < 0.001)	−14 (3.1) (*P* < 0.001)	*P* = 0.8	−11 (1.7) (*P* < 0.001)	−19 (3.2) (*P* < 0.001)	—
Primary outcomes								
ACR Pedi 90, %	One year	42%	46%	31%	—	36%	54%	—
Unadjusted, OR (95% CI)	—	—	2.0 (0.6, 6.6)	Reference.	*P* = 0.3	0.5 (0.2, 1.5)	Reference.	*P* = 0.2
Propensity adjusted^b^, OR (95% CI)	—	—	1.9 (0.4, 7.8)	Reference.	*P* = 0.4	—	—	—
Minimal disease activity, %	One year	51%	52%	49%	—	48%	58%	—
Unadjusted, OR (95% CI)	—	—	1.1 (0.4, 3.5)	Reference.	*P* = 0.8	0.7 (0.2, 2.0)	Reference.	*P* = 0.5
Propensity adjusted^b^, OR (95% CI)	—	—	1.1 (0.3, 4.3)	Reference.	*P* = 0.9	—	—	—
Clinically inactive disease, %	One year	39%	45%	25%	—	34%	52%	—
Unadjusted, OR (95% CI)	—	—	2.5 (0.8, 8.2)	Reference.	*P* = 0.1	0.5 (0.2, 1.4)	Reference.	*P* = 0.2
Propensity adjusted^b^, OR (95% CI)	—	—	2.7 (0.6, 11.2)	Reference.	*P* = 0.2	—	—	—

Using imputed data. Results displayed as mean (standard error), unless otherwise stated.

a Change in variable taking into account baseline variable (*P*-values indicates change from baseline to one year).

b Propensity score included: first biologic, gender, age, disease duration, concomitant methotrexate, concomitant steroids, active joint count, limited joint count, physician global assessment of overall disease activity (PGA), patient (parent) assessment of overall well-being (PGE), Childhood HAQ (CHAQ), ESR, JADAS-71.

JADAS-71:71-joint Juvenile Arthritis Disease Activity Score; ACR Pedi 90: ACR Paediatric criteria for 90% improvement; OR: odds ratio.

**Fig. 1:**
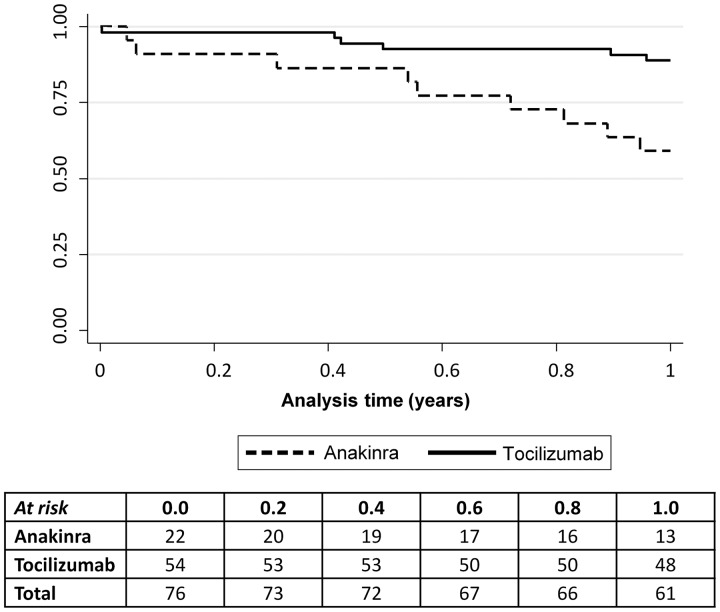
Treatment survival curve for 76 systemic JIA patients: 54 tocilizumab and 22 anakinra Survival was better on tocilizumab (89%; solid line) compared with anakinra (59%; dashed line) at one year (*P* = 0.002).

**Fig. 2:**
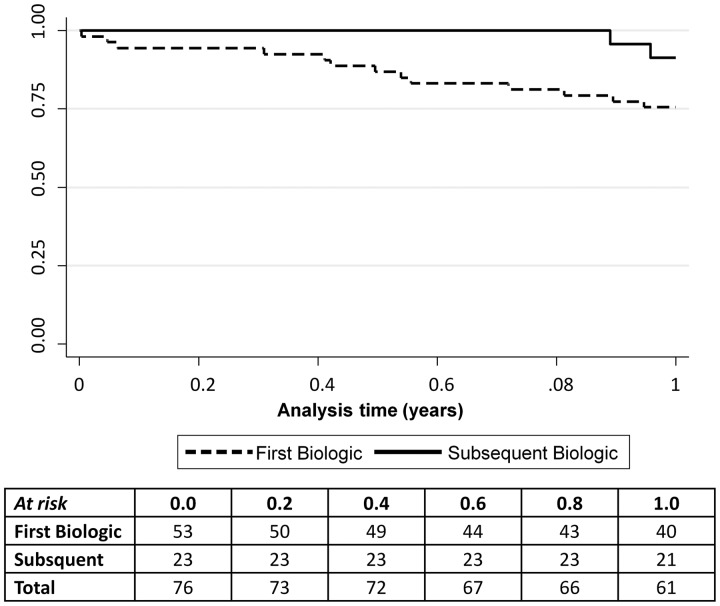
Treatment survival curve for 76 systemic JIA patients: 53 first-line and 23 subsequent biologic A slight trend towards better survival on subsequent biologic (91%; solid line) compared with first biologic use (75%; dashed line) at one year (*P* = 0.1).

Fifteen (20%) of the 76 patients stopped their prescribed biologic by one year. Treatment survival was better with tocilizumab (89%) compared with anakinra (59%; *P* = 0.002) (Fig. 1). In addition, there was a trend towards better treatment survival observed in patients starting their first biologic (91% *vs* 75%), although this was not significant (*P* = 0.1) (Fig. 2). One patient stopped due to remission (anakinra), six stopped due to inefficacy (four anakinra, two tocilizumab), seven patients stopped due to adverse events (three tocilizumab [rash worse post drug, neutropenia, active MAS (patient switched to anakinra)], four anakinra [stomach cramps and diarrhoea, injection site reaction (patient switched to etanercept), difficulty with daily injection (*n* = 2; both patients switched to tocilizumab)]), and one stopped for unknown reasons (tocilizumab).

## Discussion

This real-world, prospective, national study has demonstrated that patients with systemic JIA starting anakinra have similar response after one year compared with those starting tocilizumab, with regard to disease activity, function and outcome measures. Overall, approximately half of the patients with systemic JIA achieved a minimal disease state, and two-fifths achieved either a significant clinical short-term response or inactive disease by one year. There was no difference in effectiveness between patients treated with tocilizumab or anakinra. In addition, the proportion of patients with systemic features at one year had reduced from baseline. In the univariable analysis, none of the baseline characteristics were associated with achieving any of the three primary outcomes. One-fifth of the patients stopped biologic therapy by one year, although this was mostly due to adverse events. Treatment survival was better with tocilizumab at one year compared with anakinra, with three children stopping for anakinra injection-related problems.

It is generally reported that patients with systemic JIA have poorer outcomes compared with non-systemic JIA patients [30–36]. However, most of these studies have investigated cohorts of patients treated with TNFi that do not target the pro-inflammatory cytokines typically associated with systemic JIA [5]. One previous open label study of 112 systemic JIA patients on tocilizumab found 59% achieved an ACR Pedi 90 response at one year [14]. Whereas, data from the German biologics register BIKER found 27% of the 44 tocilizumab patients, and 35% of the 36 patients treated with an IL-1 inhibitor (either anakinra or canakinumab) achieved an ACR Pedi 90 response at one year [22]. A French retrospective study of 77 systemic JIA patients starting a first biologic (predominantly anakinra) found approximately half achieved inactive disease [17]. These proportions were similar to that seen in this current prospective observational study. In addition, only the German BIKER study compared effectiveness between IL-1 and IL-6 inhibitors and reported no statistical difference between the two drug cohorts [22].

Anakinra is currently (2015 onwards) recommended as first-line therapy for patients with active MAS, as it may help recovery [10]. However, in this study, only five of the 19 patients starting anakinra as a first-line biologic had a reported history of MAS, although this was much higher than patients starting tocilizumab. Further reasons why anakinra was selected first-line instead of tocilizumab in the remaining 11 patients was unclear. It is possible that concerns about MAS may have influenced treatment choice in some, but additional research is needed to explore this further.

In this study, almost all patients received methotrexate prior to starting tocilizumab or anakinra, in keeping with current UK guidelines [10], with a median time to first biologic of one year, less for patients starting anakinra *vs* tocilizumab. In part, this may relate to the fact that in three patients (only one with a history of MAS), first-line anakinra was used with no prior methotrexate. There is also some evidence that suggests that there may be a window of opportunity that very early use of anakinra is associated with better outcomes [19, 20], although based on the nature of this current cohort, this could not be tested further. Among this cohort, disease duration was not a predictor of a good clinical response.

At one year, 80% of systemic JIA patients in this study remained on biologic therapy. This is slightly lower compared with other studies of systemic JIA patients treated with either tocilizumab [15] or anakinra [6, 18]. Treatment survival was worse in patients receiving anakinra, with one-third of patients who stopped anakinra reporting injection-related problems. As anakinra is a daily subcutaneous injection, this has the potential to cause both physical and mental stress to the patient and/or their families. In contrast, all patients receiving tocilizumab in this study received it intravenously. Although it is important to note that fortnightly intravenous injections of tocilizumab may also be a great burden on the patients and their families.

The BCRD study represents one of the largest national cohorts of patients with JIA starting a non-Enbrel biologic. Registration into the study is highly encouraged by National Health Service England, as all patients starting a biologic should be offered the option of enrolment [10]. Therefore, it is likely that the numbers reported in this analysis were representative of the UK population. As is common in observational datasets, missing data were noted, particularly in the recording of the core outcome variables despite proactive data follow-up and capture across sites to minimise missing data. We used standardised, multiple imputation to account for this, as in all children we had information on at least some aspects of disease activity at each time point. By using multiple primary outcome measures within this analysis, the aim was to make a more generalised estimate of patient response to treatment. The ACR paediatric response criteria were initially created for non-systemic JIA patients to use in clinical trials [27]. Consequently adapting the criteria to patients with potentially very few joints affected within an observational study is challenging. The adaptations noted in the methods aimed to highlight that those patients improving their core outcome variables to zero had a positive response, and patients worsening from zero at baseline were non-responders. This remains in line with the methods from the German BIKER register [22]. A propensity score was used to balance any observed differences in patients starting the two biologic therapies. However, it is important to note that this will not account for any unmeasured confounding between the two therapies. Until this is replicated in greater patient numbers, we should remain cautious of the comparable effectiveness of tocilizumab and anakinra. While no associations were observed between clinical features and treatment response outcomes at one year, replicating the results from the German BIKER register [22], statistical significance may have been limited by power. Previous studies looking at the TNFi etanercept (Enbrel) [31, 36] have also failed to find a significant number of clinical variables associated with response. However, important short-term outcomes were able to be assessed within this analysis and as the BCRD study continues to recruit patients, and those within the study spend longer under follow-up, longer-term outcomes may be investigated in the future.

In this real-world cohort of patients with systemic JIA starting tocilizumab or anakinra, approximately half achieved a minimal disease state, and two-fifths achieved either a significant clinical short-term response or inactive disease by one year. Treatment responses appeared similar between the two biologic therapies, although low numbers prevented robust comparisons. Our observations of anakinra being used first-line in some patients, despite the availability of tocilizumab, may reflect clinicians’ preferences based on clinical scenarios and this needs further exploration. This is important to address and may inform future treatment guidelines for systemic JIA.

## Supplementary Material

Supplementary Data

## References

[1] Thierry S, Fautrel B, Lemelle I, Guillemin F. Prevalence and incidence of juvenile idiopathic arthritis: a systematic review. Joint Bone Spine 2014;81:112–7. 24210707 10.1016/j.jbspin.2013.09.003

[2] Petty RE, Southwood TR, Manners P et al International League of Associations for Rheumatology classification of juvenile idiopathic arthritis: second revision, Edmonton, 2001. J Rheumatol 2004;31:390–2. 14760812

[3] Foster H, Brogan P. Paediatric rheumatology, 1st edn Oxford: Oxford University Press, 2012.

[4] Ombrello MJ, Arthur VL, Remmers EF et al Genetic architecture distinguishes systemic juvenile idiopathic arthritis from other forms of juvenile idiopathic arthritis: clinical and therapeutic implications. Ann Rheum Dis 2017;76:906–13. 27927641 10.1136/annrheumdis-2016-210324 PMC5530341

[5] Lin YT, Wang CT, Gershwin ME, Chiang BL. The pathogenesis of oligoarticular/polyarticular vs systemic juvenile idiopathic arthritis. Autoimmun Rev 2011;10:482–9. 21320644 10.1016/j.autrev.2011.02.001

[6] Quartier P, Allantaz F, Cimaz R et al A multicentre, randomised, double-blind, placebo-controlled trial with the interleukin-1 receptor antagonist anakinra in patients with systemic-onset juvenile idiopathic arthritis (ANAJIS trial). Ann Rheum Dis 2011;70:747–54. 21173013 10.1136/ard.2010.134254 PMC3070271

[7] Yokota S, Imagawa T, Mori M et al Efficacy and safety of tocilizumab in patients with systemic-onset juvenile idiopathic arthritis: a randomised, double-blind, placebo-controlled, withdrawal phase III trial. Lancet 2008;371:998–1006. 18358927 10.1016/S0140-6736(08)60454-7

[8] Grevich S, Shenoi S. Update on the management of systemic juvenile idiopathic arthritis and role of IL-1 and IL-6 inhibition. Adolesc Health Med Ther 2017;8:125–35. 29184458 10.2147/AHMT.S109495 PMC5687245

[9] NICE. TA35: Guidance on the Use of Etanercept for the Treatment of Juvenile Idiopathic Arthritis. 2002.

[10] NHS England. Clinical Commissioning Policy Statement: Biologic Therapies for the treatment of Juvenile Idiopathic Arthritis (JIA). 2015 Contract No.: NHS England E03X04 E03/P/d Biologics for Juvenile Idiopathic Arthritis in Children and Adults.

[11] Kearsley-Fleet L, Davies R, Baildam E et al Factors associated with choice of biologic among children with Juvenile Idiopathic Arthritis: results from two UK paediatric biologic registers. Rheumatology 2016;55:1556–65. 26732349 10.1093/rheumatology/kev429 PMC4993954

[12] EMA. CHMP post-authorisation summary of positive opinion for RoActemra. European Medicines Agency, 2011 EMEA/CHMP/288328/2011.

[13] NICE. TA238: Tocilizumab for the treatment of systemic juvenile idiopathic arthritis. National Institue for Health and Care Excellence, 2011 Technology Appraisal Guidance: TA238.

[14] De Benedetti F, Brunner HI, Ruperto N et al Randomized trial of tocilizumab in systemic juvenile idiopathic arthritis. N Engl J Med 2012;367:2385–95. 23252525 10.1056/NEJMoa1112802

[15] Yokota S, Itoh Y, Morio T et al Tocilizumab in systemic juvenile idiopathic arthritis in a real-world clinical setting: results from 1 year of postmarketing surveillance follow-up of 417 patients in Japan. Ann Rheum Dis 2016;75:1654–60. 26644233 10.1136/annrheumdis-2015-207818 PMC5013079

[16] Pacharapakornpong T, Vallibhakara SA, Lerkvaleekul B, Vilaiyuk S. Comparisons of the outcomes between early and late tocilizumab treatment in systemic juvenile idiopathic arthritis. Rheumatol Int 2017;37:251–5. 27798725 10.1007/s00296-016-3595-z

[17] Woerner A, Uettwiller F, Melki I et al Biological treatment in systemic juvenile idiopathic arthritis: achievement of inactive disease or clinical remission on a first, second or third biological agent. RMD Open 2015;1:e000036. 26509061 10.1136/rmdopen-2014-000036 PMC4613174

[18] Lequerré T, Quartier P, Rosellini D et al Interleukin-1 receptor antagonist (anakinra) treatment in patients with systemic-onset juvenile idiopathic arthritis or adult onset Still disease: preliminary experience in France. Ann Rheum Dis 2007;67:302–8. 17947302 10.1136/ard.2007.076034

[19] Nigrovic PA, Mannion M, Prince FH et al Anakinra as first-line disease-modifying therapy in systemic juvenile idiopathic arthritis: report of forty-six patients from an international multicenter series. Arthritis Rheum 2011;63:545–55. 21280009 10.1002/art.30128

[20] Vastert SJ, de Jager W, Noordman BJ et al Effectiveness of first-line treatment with recombinant interleukin-1 receptor antagonist in steroid-naive patients with new-onset systemic juvenile idiopathic arthritis: results of a prospective cohort study. Arthritis Rheumatol 2014;66:1034–43. 24757154 10.1002/art.38296

[21] Pardeo M, Pires Marafon D, Insalaco A et al Anakinra in systemic juvenile idiopathic arthritis: a single-center experience. J Rheumatol 2015;42:1523–7. 26034148 10.3899/jrheum.141567

[22] Horneff G, Schulz AC, Klotsche J et al Experience with etanercept, tocilizumab and interleukin-1 inhibitors in systemic onset juvenile idiopathic arthritis patients from the BIKER registry. Arthritis Res Ther 2017;19:256. 29166924 10.1186/s13075-017-1462-2 PMC5700562

[23] Davies R, Southwood TR, Kearsley-Fleet L et al Medically significant infections are increased in patients with juvenile idiopathic arthritis treated with etanercept: results from the British Society for Paediatric and Adolescent Rheumatology Etanercept Cohort Study. Arthritis Rheumatol 2015;67:2487–94. 25989609 10.1002/art.39197 PMC5049649

[24] Consolaro A, Ruperto N, Bazso A et al Development and validation of a composite disease activity score for juvenile idiopathic arthritis. Arthritis Rheum 2009;61:658–66. 19405003 10.1002/art.24516

[25] Magni-Manzoni S, Ruperto N, Pistorio A et al Development and validation of a preliminary definition of minimal disease activity in patients with juvenile idiopathic arthritis. Arthritis Rheum 2008;59:1120–7. 10.1002/art.23916 18668599

[26] Wallace CA, Ruperto N, Giannini E et al Preliminary criteria for clinical remission for select categories of juvenile idiopathic arthritis. J Rheumatol 2004;31:2290–4. 15517647

[27] Giannini EH, Ruperto N, Ravelli A et al Preliminary definition of improvement in juvenile arthritis. Arthritis Rheum 1997;40:1202–9. [9214419] 9214419 10.1002/1529-0131(199707)40:7<1202::AID-ART3>3.0.CO;2-R

[28] White IR, Royston P, Wood AM. Multiple imputation using chained equations: issues and guidance for practice. Stat Med 2011;30:377–99. 21225900 10.1002/sim.4067

[29] StataCorp. Stata statistical software: release 13. College Station, TX: StataCorp LP, 2013.

[30] Quartier P, Taupin P, Bourdeaut F et al Efficacy of etanercept for the treatment of juvenile idiopathic arthritis according to the onset type. Arthritis Rheuma 2003;48:1093–101. 10.1002/art.10885 12687553

[31] Otten MH, Prince FH, Armbrust W et al Factors associated with treatment response to etanercept in juvenile idiopathic arthritis. JAMA 2011;306:2340–7. 22056397 10.1001/jama.2011.1671

[32] Papsdorf V, Horneff G. Complete control of disease activity and remission induced by treatment with etanercept in juvenile idiopathic arthritis. Rheumatology(Oxford) 2011;50:214–21. 21148155 10.1093/rheumatology/keq292

[33] Zuber Z, Rutkowska-Sak L, Postepski J et al Etanercept treatment in juvenile idiopathic arthritis: the Polish registry. Med Sci Monit 2011;17:SR35–42. 22129916 10.12659/MSM.882109 PMC3628139

[34] Solari N, Palmisani E, Consolaro A et al Factors associated with achievement of inactive disease in children with juvenile idiopathic arthritis treated with etanercept. J Rheumatol 2013;40:192–200. 23204218 10.3899/jrheum.120842

[35] Geikowski T, Becker I, Horneff G, German BIKER Registry Collaborative Study Group. Predictors of response to etanercept in polyarticular-course juvenile idiopathic arthritis. Rheumatology 2014;53:1245–9. 24599916 10.1093/rheumatology/ket490

[36] Kearsley-Fleet L, Davies R, Lunt M, Southwood TR, Hyrich KL. Factors associated with improvement in disease activity following initiation of etanercept in children and young people with Juvenile Idiopathic Arthritis: results from the British Society for Paediatric and Adolescent Rheumatology Etanercept Cohort Study. Rheumatology 2016;55:840–7. 26721878 10.1093/rheumatology/kev434 PMC4830911

