# Supplementary material for: Short-term outcomes in patients with systemic juvenile idiopathic arthritis treated with either tocilizumab or anakinra
Source: Rheumatology (Oxford). 2018 Aug 21;58(1):94–102. doi: 10.1093/rheumatology/key262 (PMC6293481; doi:10.1093/rheumatology/key262)
Supplement: Supplementary Data [file key262_suppl_data.docx]

**SUPPLEMENTARY DATA**

**Supplementary Table S1: Odds ratios for each primary outcome with baseline characteristics measured at start of biologic**

|  | Odds Ratio for ACR Paediatric 90% response (ACR Pedi 90) | Odds Ratio for Minimal Disease Activity (MDA) | Odds Ratio for Clinically Inactive Disease (CID) |
| --- | --- | --- | --- |
| Tocilizumab (vs. anakinra) | 2.0 (0.6, 6.6) p=0.3 | 1.1 (0.4, 3.5) p=0.8 | 2.5 (0.8, 8.2) p=0.1 |
| First Biologic (vs. subsequent) | 0.5 (0.2, 1.5) p=0.2 | 0.7 (0.2, 2.0) p=0.5 | 0.5 (0.2, 1.4) p=0.2 |
| Female (vs. Male) | 0.9 (0.3, 2.5) p=0.8 | 0.9 (0.3, 2.4) p=0.8 | 0.9 (0.3, 2.6) p=0.9 |
| Age, years | 1.0 (0.9, 1.2) p=0.5 | 1.1 (0.9, 1.2) p=0.4 | 1.1 (1.0, 1.2) p=0.3 |
| Disease Duration , years | 1.1 (0.8, 1.3) p=0.6 | 1.2 (0.9, 1.6) p=0.1 | 1.1 (0.9, 1.4) p=0.4 |
| Methotrexate use (vs. none) | 1.1 (0.3, 4.3) p=0.9 | 1.4 (0.4, 5.5) p=0.6 | 0.5 (0.1, 2.4) p=0.4 |
| Oral steroid use (vs. none) | 1.0 (0.3, 2.9) p=1.0 | 0.6 (0.2, 1.8) p=0.4 | 1.0 (0.4, 2.9) p=1.0 |
| Active Joint Count, joints | 1.0 (0.9, 1.1) p=0.8 | 1.0 (0.9, 1.0) p=0.2 | 1.0 (0.9, 1.0) p=0.2 |
| Limited Joint Count, joints | 1.0 (0.9, 1.1) p=0.7 | 1.0 (0.9, 1.1) p=0.8 | 1.0 (0.9, 1.0) p=0.3 |
| PGA, cms | 1.1 (0.9, 1.3) p=0.6 | 0.9 (0.7, 1.1) p=0.3 | 0.9 (0.7, 1.1) p=0.4 |
| PGE, cms | 1.1 (0.9, 1.3) p=0.3 | 0.9 (0.8, 1.1) p=0.5 | 1.0 (0.8, 1.2) p=1.0 |
| CHAQ (whole unit) | 1.0 (0.5, 2.0) p=0.9 | 0.7 (0.3, 1.4) p=0.3 | 0.7 (0.4, 1.4) p=0.3 |
| ESR, mm/hr | 1.0 (1.0, 1.0) p=0.3 | 1.0 (1.0, 1.0) p=0.9 | 1.0 (1.0, 1.0) p=0.7 |
| JADAS-71 | 1.0 (1.0, 1.1) p=0.6 | 1.0 (0.9, 1.0) p=0.2 | 1.0 (0.9, 1.0) p=0.4 |

Using imputed data. American College of Rheumatology (ACR), Physician Global Assessment of disease (PGA), Parent (or Parent) Global Evaluation of well-being (PGE), Childhood Health Assessment Questionnaire (CHAQ), Erythrocyte Sedimentation Rate (ESR), 71-joint Juvenile Arthritis Disease Activity Score (JADAS-71).
